# Pain, mood, and suicidal behavior among injured working adults in Chile

**DOI:** 10.1186/s12888-022-04391-3

**Published:** 2022-12-06

**Authors:** Juan Carlos Vélez, Michael Kovasala, Michele Demi Collado, Lauren E. Friedman, Diana L. Juvinao-Quintero, Lisette Araya, Jessica Castillo, Michelle A. Williams, Bizu Gelaye

**Affiliations:** 1 Departamento de Rehabilitación, Hospital del Trabajador, Santiago, Chile; 2 Multidisciplinary International Research Training (MIRT) Program, Harvard T.H. Chan School of Public Health, Boston, MA USA; 3 Department of Epidemiology, Harvard T.H. Chan School of Public Health, 677 Huntington Ave Room 505F, Boston, MA 02115 USA; 4 Servicio de Psiquiatría y Psicología, Hospital del Trabajador, Santiago, Chile; 5 The Chester M. Pierce, MD Division of Global Psychiatry, Massachusetts General Hospital, Boston, MA USA; 6 Department of Psychiatry, Harvard Medical School, Boston, MA USA

**Keywords:** Pain, Chronic pain, Depression, Anxiety, Suicidal ideation, Suicidal behavior, Injured working adults

## Abstract

**Background:**

Chronic pain is comorbid with psychiatric disorders, but information on the association of chronic pain with depressive symptoms, generalized anxiety, and suicidal behavior among occupational cohorts is inadequate. We investigated these associations among employed Chilean adults.

**Methods:**

A total of 1946 working adults were interviewed during their outpatient visit. Pain was assessed using the Short Form McGill Pain questionnaire (SF-MPG) while depression and generalized anxiety were examined using the Patient Health Questionnaire-9 (PHQ-9) and Generalized Anxiety Disorder-7 (GAD-7), respectively. The Columbia–Suicide Severity Rating Scale was used to assess suicidal behavior and suicidal ideation. Multivariable logistic regression models were used to estimate adjusted odds ratios (aORs) and 95% confidence intervals (95%CI) for the association of chronic pain with mood disorders, as well as suicidal behavior.

**Results:**

High chronic pain (SF-MPG > 11) was reported by 46% of participants. Approximately two-fifths of the study participants (38.2%) had depression, 23.8% generalized anxiety, 13.4% suicidal ideation, and 2.4% suicidal behavior. Compared to those with low pain (SF-MPG ≤11), participants with high chronic pain (SF-MPG > 11) had increased odds of experiencing depression only (aOR = 2.87; 95% CI: 2.21–3.73), generalized anxiety only (aOR = 2.38; 95% CI: 1.42–3.99), and comorbid depression and generalized anxiety (aOR = 6.91; 95% CI: 5.20–9.19). The corresponding aOR (95%CI) for suicidal ideation and suicidal behavior were (aOR = 2.20; 95% CI: 1.58–3.07) and (aOR = 2.18 = 95% CI: 0.99–4.79), respectively.

**Conclusions:**

Chronic pain is associated with increased odds of depression, generalized anxiety, and suicidal behavior. Mental health support and appropriate management of patients experiencing chronic pain are critical.

**Supplementary Information:**

The online version contains supplementary material available at 10.1186/s12888-022-04391-3.

## Introduction

Chronic pain is a debilitating health condition estimated to affect 1 in 5 adults worldwide [1] with profound emotional and cognitive effects [2] and economic impacts, including loss of productivity, absenteeism due to illness, presenteeism, and loss of employment [3]. In the United States alone, 100 million adults — more than the number affected by heart disease, diabetes, and cancer combined — suffer from common chronic pain conditions, with an estimated loss in productivity due to chronic pain being $61 billion per year [4]. Several epidemiologic studies have shown associations between chronic pain and mood disorders, including generalized anxiety and depression [5–8]. More than half of people treated for depression also report problems with pain [9]. A growing body of evidence shows patients suffering from chronic physical pain are more likely to report suicidal behavior, ideation, plans, and/or attempts [10–14]. Of note, patients with chronic pain are at an increased risk of dying from suicide compared to those without chronic pain [13, 14]. Few previous studies have examined the association between chronic pain and depression and generalized anxiety in South America, including in Colombia [8, 15], Brazil [7, 16, 17], Paraguay [7] and Ecuador [7]. Only two of these previous studies have examined the association between pain and suicidal behaviors [18, 19], with one study reporting null findings [19].

Given that (1) few studies have examined pain with depression, generalized anxiety, and suicidal behaviors in South American populations; (2) one previous study found no association between pain and suicide risk in Colombia [19], and (3) no study has been conducted in Chile, the most stable and prosperous country in South America, we conducted a larger-scale cross-sectional epidemiological study to investigate the association of chronic pain with symptoms of depression, generalized anxiety, and suicidal behavior among injured working adults in Chile.

## Materials and methods

### Study population

The sample for this study included participants of the Stress, Pain, Sleep, and Neuropsychiatric Disorders (SPLENDID) study in a workers’ compensation hospital system in Chile. The SPLENDID study was designed to examine pain, work-related stress, and neuropsychiatric outcomes in the context of injured working adults in Chile with the intention of developing workplace intervention programs. The study was conducted between September 2015 and February 2018 among patients attending the Hospital del Trabajador in Santiago, Chile. The Hospital del Trabajador is the largest workers’ compensation hospital and the referral center for trauma and professional diseases of Asociación Chilena de Seguridad, the biggest workers’ compensation system in Chile, with almost 2.5 million affiliated workers. Working adults were eligible for the study if they attended the workers’ compensation hospital for the following injuries: spinal cord, mild brain injury, bone fractures, burns, and soft tissue injuries of various etiologies. Eligible participants also must be able to read and write Spanish. All participants provided written informed consent. The institutional review boards of the Hospital del Trabajador, Santiago, Chile, and the Office of Human Research Administration, Harvard T.H. Chan School of Public Health, Boston, MA, approved all procedures used in this study.

#### Analytic population

A total of 2000 participants were interviewed. Of these, 21 participants were excluded due to missing information on the Short Form McGill Pain Questionnaire, 4 were excluded for missing information on the Patient Health Questionnaire-9, 8 were excluded for missing information on the Generalized Anxiety Disorder-7, 9 were excluded for missing data on CSSRS suicidal ideation, 22 were excluded for missing data on CSSRS suicidal behavior, and 1 participant was excluded for being outside the age range (> 85). A total of 1946 participants were included in the final analysis.

### Study procedures

#### Pain assessment

The Short-Form McGill Pain Questionnaire (SF-MPG) was used to measure current perceived pain among adults with chronic pain [20]. The SF-MPG comprises two subscales: a sensory subscale with 11 words or items and an affective subscale with 4 words or items. The sensory subscale includes items that describe the properties of the pain experienced (i.e., continuous, intermittent or neuropathic pain), whereas the affective subscale includes items that describe the emotional perception of pain (i.e., tiring-exhausting, sickening, fearful, and punishing-cruel) [21]. The items are rated on a Likert Scale for pain intensity with 0 = none, 1 = mild, 2 = moderate, and 3 = severe. The total score is obtained by summing the item scores. The sensory subscale total score ranges from 0 to 33, and affective subscale total score ranges from 0 to 12. The SF-MPG total overall score ranges from 0 to 45. We used the median score of the two subscales to define high and low pain scores (SF-MPG median: 11.0, SF-MPG sensory subscale median: 9.0, SF-MPG affective subscale median: 2.0). This approach is consistent with prior studies that summarized pain scores based on their distribution in the absence of a clinical diagnostic cutoff [22, 23]. In addition, the median split is a common method used to dichotomize variables in psychological research [24, 25], and in other fields [26], and oftentimes it is viewed as a more parsimonious way to split continuous data [24]. In addition, the cut-off we used to group between low and high pain in our study was consistent with mean scores [27, 28] and median scores [29] reported in the literature for the SF-MPG.

#### Depressive symptoms assessment

The Patient Health Questionnaire-9 (PHQ-9) was used to screen participants for depression. The PHQ-9 contains 9 questions about a participants’ depression symptoms over the 14 days prior to assessment. The items were scored from 0 to 3 according to the response categories: never, several days, more than half the days, or nearly every day. The total PHQ-9 score ranged from 0 to 27, and presence of depressive symptoms was defined as a PHQ-9 score ≥ 10 based on the accepted diagnostic properties for depression at this cut-off [30]. The PHQ-9 was previously validated in Spanish-speaking populations [31, 32] and specifically in the Chilean population [33].

#### Generalized anxiety symptoms assessment

Generalized anxiety symptoms was assessed using the Generalized Anxiety Disorder Scale (GAD-7). The GAD-7 contains seven questions about generalized anxiety symptoms over the 14 days prior to assessment. Items were scored from 0 to 3 according to response categories: never, several days, more than half the days, or nearly every day. The total score ranges from 0 to 21. The presence of anxiety symptoms was defined as a GAD-7 score ≥ 10, being this a reasonable cut-point to identify moderate to severe cases of GAD [34]. The GAD-7 has previously been used in Spanish-speaking populations [35, 36].

#### Suicidal assessment

Suicidal ideation and suicidal behaviors during the past month were assessed using the Columbia Suicidal Rating Scale (C-SSRS) Screen version [37, 38]. The C-SSRS Screen is a structured interview based on the more comprehensive full-length version. The scale measures suicidal ideation severity and suicidal behavior using two subsets of items. The first subset captures suicidal ideation severity during the past month. The questions ask: wish to be dead, non-specific active suicidal thoughts, suicidal thoughts with methods, suicidal intent, and suicidal intent with plan. The second subset measures suicidal behavior during the past three months. The questions asked about the presence of actual and aborted suicide attempts. For the purposes of the present study, suicidal ideation was defined by a “yes” answer to any one of the five suicidal ideation severity items. Suicidal behavior was defined as a “yes” answer to the suicidal behavior question. The C-SSRS has previously been validated in Spanish-speaking populations [39, 40].

#### Covariates

Structured questionnaires were used to determine participants’ sociodemographic and occupational characteristics. Participants age was categorized as 18–24, 25–34, 35–44, 45–54, 55–64, 65–74, and 75–84 years. Other sociodemographic characteristics examined were sex (male vs. female), country of birth (Chile vs. others), belonging to indigenous or native groups (no vs. yes), highest degree of education attained (elementary school, high school, college or technical training), marital status (married/living with a partner, single, previously married), body mass index (BMI; < 18.5, 18.5–24.9, 25–29.9, > 30 kg/m^2^), lifetime smoking (no vs. yes), and lifetime alcohol consumption (no vs. yes). Participants’ occupational characteristics included work sector (construction, finance, commercial, manufacturing, public services, transportation, others), type of occupation (administrative, manual worker, professional, salesperson, technician, teacher, others), time since the accident in days (1–42 days: Acute; 43–84 days: Subacute; > 84 days: Chronic) [41, 42], type of accident (commute or work injury), and type of injury (burn, fall, cut, attrition, firearm, blunt trauma, repetitive use, others) [43].

### Statistical analysis

Frequency distributions of sociodemographic and occupational characteristics were examined using mean ± standard deviation (SD) for continuous variables and numbers and percentages (%) for categorical variables. Associations between pain status and sociodemographic and occupational characteristics were examined using Chi-squared tests for categorical variables and analysis of variance (ANOVA) for continuous variables. The median value [25th and 75th percentiles] was calculated for time since the accident, as the distribution of these values was right-skewed. Wilcoxon-Mann Whitney test was used to evaluate differences in medians. PHQ-9 and GAD-7 scores were used to create categorical variables: depression or anxiety symptoms (no vs. yes); and mood disorder status (no depression or anxiety, depression only, anxiety only, or both depression and anxiety). Multivariable logistic regression procedures were used to calculate odds ratios (ORs) and 95% confidence intervals (95% CI) for the association of chronic pain with depressive symptoms, generalized anxiety symptoms, and suicidal behaviors. We fit separate models with symptoms of depression, generalized anxiety and suicidal behaviors as outcome and chronic pain as exposure. Assessment of confounders was performed by entering a priori selected putative confounders (e.g., age, sex, marital status, type of workplace and type of injury) [44–46] into a logistic regression model one at a time and by comparing the adjusted OR (aOR) and unadjusted ORs. Final logistic regression models included covariates that altered unadjusted ORs by at least 10%. Additionally, the suicidal ideation and behavior models included further adjustment for depression status as measured by the first eight questions of the PHQ-9 [46, 47]. Question 9 was not included since it asks about suicidal ideation (‘Thoughts that you would be better off dead, or of hurting yourself in some way’). The PHQ-8 depression questionnaire has been demonstrated to minimally influence scale performance, mean scores, or diagnostic cut points compared with the PHQ-9 [48, 49]. Statistical analyses were performed using SPSS Statistics, Version 23.0 (IBM SPSS v23.0, Armonk, NY, USA).

## Results

Sociodemographic characteristics of the study population are shown in Table 1. The mean age of participants was 45.9 ± 13.7 years old. Participants were more likely to be men (72.9%) and born in Chile (94.1%). Only 3.2% of participants self-identified as belonging to an indigenous or native group. Most participants had at least a high school or some college education (83.9%), were married or living with a partner (62.1%) and had a lifetime history of smoking (58.4%) and alcohol consumption (61.8%). Approximately 46% of participants were classified as having high perceived chronic pain as measured by the SF-MPG. Participants with high pain were more likely to be older, men, and be more highly educated (*p* < 0.001; Table 1).

**Table 1:** Sociodemographic characteristics of a population of injured working adults in Santiago, Chile according to pain status as measured by the Short-Form McGill Pain Questionnaire ^a^ (*N* = 1946)

Characteristics	All participants (***N*** = 1946)		Low pain (***N*** = 1053)		High pain (***N*** = 893)		***P***-value
	n	%	n	%	n	%	
Age (years), mean ± SD ^b^	45.9 ± 13.7		44.6 ± 14.2		47.4 ± 12.9		**< 0.001**
18–24	141	7.2	97	9.2	44	4.9	**< 0.001**
25–34	347	17.8	213	20.2	134	15.0	
35–44	366	18.8	207	19.7	159	17.8	
45–54	494	25.4	237	22.5	257	28.8	
55–64	452	23.2	219	20.8	233	26.1	
65–74	129	6.6	71	6.7	58	6.5	
75–84	17	0.9	9	0.9	8	0.9	
Sex							
Men	1418	72.9	842	80.0	576	64.5	**< 0.001**
Women	528	27.1	211	20.0	317	35.5	
Country of birth							
Chile	1832	94.1	981	93.2	851	95.3	**0.046**
Other	114	5.9	72	6.8	42	4.7	
Belong to indigenous/native group							
No	1883	96.8	1021	97.0	862	96.6	0.685
Yes	62	3.2	32	3.0	30	3.4	
Highest degree of education							
Elementary school	313	16.1	150	14.3	163	18.3	**0.002**
High school	1066	54.8	615	58.5	451	50.5	
College or technical training	566	29.1	287	27.3	279	31.2	
Marital Status							
Married/living with a partner	1207	62.1	681	64.7	526	58.9	**< 0.001**
Single	464	23.9	259	24.6	205	23.0	
Previously married ^c^	274	14.1	112	10.6	162	18.1	
Body Mass Index (kg/m^2^)							
< 18.5	12	0.6	7	0.7	5	0.6	0.291
18.5–24.9	468	24.1	263	25.0	205	23.1	
25–29.9	842	43.4	466	44.3	376	42.3	
> 30	617	31.8	315	30.0	302	34.0	
Lifetime smoking							
No	810	41.6	442	42.0	368	41.2	0.733
Yes	1136	58.4	611	58.0	525	58.8	
Current smoking							
No	1417	72.8	799	75.9	618	69.2	**0.001**
Yes	529	27.2	254	24.1	275	30.8	
Lifetime alcohol consumption							
No	744	38.2	363	34.5	381	42.7	**< 0.001**
Yes	1202	61.8	690	65.5	512	57.3	
Current alcohol consumption							
No	739	38.0	358	34.0	381	42.7	**< 0.001**
Yes	1206	62.0	695	66.0	511	57.3	

^a^Categorical variable for SF-MPG total score uses a median split with low reported pain categorized as below the median and high pain as above the median (SF-MPG median: 11.0)

^b^Based on reported age

^c^Widowed, separated, or divorced

Participants worked in the sectors of manufacturing (26.6%) or public services (21.4%). The majority of participants were most likely to classify their occupation as manual workers (58.1%). The median time since their accident was 188 days. The majority of accidents occurred during work (65.6%) and were attributed to falling (35.5%) or blunt trauma (32.6%). Participants with high pain were less likely to be manual workers, reported a longer time since the accident occurred, and were also more likely to have falling or blunt trauma injuries (*p* < 0.001; Table 2).

**Table 2:** Occupation and injury characteristics of a working adult population in Santiago, Chile according to pain status as measured by the Short-Form McGill Pain Questionnaire ^a^ (*N* = 1946)

Characteristics	All participants (***N*** = 1946)		Low pain (***N*** = 1053)		High pain (***N*** = 893)		***P***-value
	n	%	n	%	n	%	
***Occupation***							
Work sector							
Construction	217	11.2	134	12.7	83	9.3	**< 0.001**
Finances	36	1.8	23	2.2	13	1.5	
Commercial	356	18.3	222	21.1	134	15.0	
Manufacturing	518	26.6	294	27.9	224	25.1	
Public services	417	21.4	188	17.9	229	25.6	
Transportation	175	9.0	104	9.9	71	8.0	
Other^b^	227	11.7	88	8.4	139	15.6	
Type of occupation							
Administrative	262	13.5	152	14.4	110	12.3	**< 0.001**
Manual worker	1130	58.1	662	62.9	468	52.5	
Professional	130	6.7	68	6.5	62	7.0	
Salesperson	52	2.7	25	2.4	27	3.0	
Technician	157	8.1	62	5.9	95	10.7	
Teacher	14	0.7	7	0.7	7	0.8	
Other^c^	200	10.3	77	7.3	123	13.8	
***Diagnosis and injury***							
Time since the accident, median [IQR] (days)^d^	188 [71–524]		153 [59–391]		245 [88–846]		**< 0.001**
Acute (1–42)	291	15.0	181	17.2	110	12.3	**< 0.001**
Subacute (43–84)	267	13.7	161	15.3	106	11.9	
Chronic (> 84)	1388	71.3	711	67.5	677	75.8	
Type of accident							
Commute	669	34.4	337	32.0	332	37.3	**0.015**
Work injury	1275	65.6	716	68.0	559	62.7	
Type of injury							
Burn	49	2.5	28	2.7	21	2.4	**< 0.001**
Fall	690	35.5	346	32.9	344	38.5	
Cut	192	9.9	134	12.7	58	6.5	
Attrition	249	12.8	159	15.1	90	10.1	
Firearm	33	1.7	27	2.6	6	0.7	
Blunt trauma	635	32.6	318	30.2	317	35.5	
Repetitive use	7	0.4	4	0.4	3	0.3	
Other	90	4.6	36	3.4	54	6.0	

^a^Categorical variable for SF-MPG total score uses a median split with low reported pain categorized as below the median and high pain as above the median (SF-MPG median: 11.0). ^b^Includes agriculture, education, security, cleaning services, administration, food service, automotive, mining, retired, gardener, electrical engineer, maintenance, etc. ^c^Includes chauffeur, conductors, concierge, security guard, food service, landlord, food distribution, telecommunication, machine operator, cleaning services, etc. ^d^Median value [25th and 75th percentiles] were calculated for time since accident, since the distribution of these values was right skewed. Wilcoxon-Mann Whitney test was used to evaluate differences in medians. Fisher’s exact test was used to assess difference between categorical variables

Psychiatric characteristics of the population are presented in Table 3. Overall, 38.2% of the population had depression symptoms, and 23.8% had generalized anxiety symptoms. 13.4% of the study population had suicidal ideation, and 2.4% had suicidal behavior. Participants with high pain were significantly more likely to have depression or generalized anxiety symptoms, suicidal ideation, and suicidal behavior (Table 3).

**Table 3:** Psychiatric characteristics of a population of injured working adults in Santiago, Chile according to pain status as measured by the Short-Form McGill Pain Questionnaire^a^ (*N* = 1946)

Characteristics	All participants (***N*** = 1946)		Low pain (***N*** = 1053)		High pain (***N*** = 893)		***P***-value
	n	%	n	%	n	%	
Depression^***b***^ or anxiety							
No	1131	58.1	778	73.9	353	39.5	**< 0.001**
Yes	815	41.9	275	26.1	540	60.5	
Depression or anxiety							
No	1131	58.1	778	73.9	353	39.5	**< 0.001**
Depression only (PHQ-9)	352	18.1	154	14.6	198	22.2	
Anxiety only (GAD-7)	71	3.6	35	3.3	36	4.0	
Both depression and anxiety	392	20.1	86	8.2	306	34.3	
***Suicidal Ideation***							
Suicidal ideation							
No suicidal ideation	1685	86.6	989	93.9	696	77.9	**< 0.001**
Suicidal ideation	261	13.4	64	6.1	197	22.1	
Suicidal behavior							
No suicidal behavior	1899	97.6	1044	99.1	855	95.7	**< 0.001**
Suicidal behavior	47	2.4	9	0.9	38	4.3	

^a^Categorical variable for SF-MPG total score uses a median split with low reported pain categorized as below the median and high pain as above the median (SF-MPG median: 11.0)

^b^Participants with PHQ-9 > 10 are characterized as having depression symptoms. Those with GAD-7 ≥ 10 are characterized has having generalized anxiety symptoms

Participants with high pain had 4.05-fold increased odds of experiencing symptoms of depression or generalized anxiety compared to those who reported low pain after adjusting for sociodemographic and occupational confounders (aOR = 4.05; 95%CI: 3.32–4.94). Compared to participants with low pain, those who reported high pain had higher odds of depression (aOR = 2.74; 95% CI: 2.13–3.52) and generalized anxiety symptoms alone (aOR = 2.16; 95% CI: 1.32–3.53), and co-occurring symptoms of depression and generalized anxiety (aOR = 7.19 95% CI: 5.45–9.51). After adjusting for putative confounders, participants with high pain had 3.48-fold increased odds of reporting suicidal ideation than those with low pain (aOR = 3.70; 95%CI: 2.72–5.04). Further adjustment for depressive symptoms attenuated this association but remained strong (aOR = 2.34; 95%CI: 1.68–3.27). Participants with high pain had 4.05-fold increased odds of reporting suicidal behavior compared to those with low pain (OR = 4.05; 95%CI: 1.92–8.54). After adjusting for symptoms of depression, the magnitude of the association between high pain and suicidal behavior was attenuated (OR = 2.35; 95%CI: 1.08–5.07; Table 4). We further conducted an additional analysis using the SF-MPG continuous score, and the interpretation remained the same, but the associations were largely attenuated (Supplementary Table 1). The associations between pain and symptoms of depression, generalized anxiety, and suicidal behaviors were similar when analyzed separately using the SF-MPG sensory pain and affective pain subscales (Supplementary Tables 2 and 3).

**Table 4:** Association of pain with depression, anxiety, and suicidal behavior among a population of injured working adults in Santiago, Chile (*N* = 1946)

Depression, anxiety and suicidal ideation	Low pain (***N*** = 1053)		High pain (***N*** = 893)					
	n	%	n	%	Unadjusted OR (95% CI)	Adjusted OR (95% CI)^**a**^	Adjusted OR (95% CI)^**b**^	Adjusted OR (95% CI)^**c**^
Depression or anxiety								
No	778	73.9	353	39.5	Reference	Reference	Reference	–
Yes	275	26.1	540	60.5	4.33 (3.57–5.24)	4.03 (3.31–4.90)	4.05 (3.32–4.94)	–
Depression and anxiety								
No	778	73.9	353	39.5	Reference	Reference	Reference	–
Depression only (PHQ-9)	154	14.6	198	22.2	2.83 (2.22–3.62)	2.73 (2.13–3.51)	2.74 (2.13–3.52)	–
Anxiety only (GAD-7)	35	3.3	36	4.0	2.27 (1.40–3.67)	2.13 (1.30–3.48)	2.16 (1.32–3.53)	–
Both depression and anxiety	86	8.2	306	34.3	7.84 (5.99–10.28)	7.05 (5.36–9.28)	7.19 (5.45–9.51)	–
***Suicidal Behaviors***								
Suicidal ideation								
No suicidal ideation	989	93.9	696	77.9	Reference	Reference	Reference	Reference
Suicidal ideation	64	6.1	197	22.1	4.37 (3.25–5.89)	4.06 (2.99–5.51)	3.70 (2.72–5.04)	2.34 (1.68–3.24)
Suicidal behavior								
No suicidal behavior	1044	99.1	855	95.7	Reference	Reference	Reference	Reference
Suicidal behavior	9	0.9	38	4.3	5.16 (2.48–10.72)	4.77 (2.27–10.02)	4.05 (1.92–8.54)	2.35 (1.08–5.07)

^a^Adjusted for age (continuous), sex (male/female), marital status (married/single/previously married)

^b^Further adjusted for type of workplace (categories), type of injury (categories), and time since the accident (categories)

^c^Further adjusted for type of workplace (categories), type of injury (categories), time since accident (categories), and depression (PHQ-8)

## Discussion

Pain is highly prevalent and contributes greatly to morbidity and mortality. In a review of 18 adult population surveys from 10 high-income countries (HICs) and seven low- or middle-income countries LMICs (*N* = 42,249), the prevalence of chronic pain was 37.3% in HICs and 41.1% in LMICs [8]. In our study of injured Chilean working adults, 46% of participants were classified as having chronic pain, and they were more likely to be older, men, current smokers, and had higher levels of education compared to participants with low pain. Our study findings and available data collectively show that chronic pain is generalized. The positive association that we observed between chronic pain and age was largely consistent with findings reported from other epidemiologic studies [50, 51]; however, the prevalence of pain across demographic characteristics such as gender and education level in our study differed from previous evidence [51], including that from a study conducted in Chile and another in Colombia [52, 53]. These differences in the prevalence of chronic pain might be attributable to differences in populations included, measurement scales used, and study designs employed.

Chronic pain was associated with depression and anxiety spectrum disorders in both LMICs and HICs [8]. In a recent review of 47 LMICs (*N* = 273,952), depression was significantly associated with severe pain in 44 of the 47 countries included (OR = 3.93; 95% CI 3.54–4.37) [7]. Neither of these prior reviews includes associations between pain and mood disorder symptoms in a Chilean population. This is important as chronic pain has been reported to be highly prevalent in 32% of the general population [52]. To the best of our knowledge, our study is the first to examine this association in a Chilean population. Taken together, our study and previous work demonstrate a robust association between chronic pain and mood disorders, including depression and anxiety.

Previous studies have examined the association between chronic pain and suicidal behaviors. Several studies conducted in the United States [54, 55], Australia [56], Canada [57], Japan [58], and India [59] have shown associations between chronic pain and suicidal ideation or behaviors. For example, among U.S. adults (*N* = 5692), the presence of a pain condition was significantly associated with lifetime suicidal ideation (OR = 1.4; 95%CI: 1.1–1.8) and plans (OR = 1.5; 95%CI: 1.1–2.1) [55]. Among Canadian adults over 66 years old, completed suicide was associated with moderate (OR = 1.91; 95% CI: 1.66–2.20) and severe pain (OR = 7.52; 95% CI: 4.93–11.46). Among patients admitted for attempted suicide and controls at a teaching hospital in India (*N* = 137 patients and *N* = 137 controls), those with idiopathic pain (OR = 6.78; 95% CI: 2.39–20.76) and physical illnesses (OR = 3.12; 95% CI: 1.37–7.24) had an increased odds of suicide attempts compared to controls [59]. To our knowledge, only one study has examined the association between pain and suicide risk in South America. In an underpowered study conducted at a hospital pain clinic in Colombia (*N* = 49), the authors found no association between the risk of suicide and perceived levels of pain [19]. In our study, participants with high pain were more likely to report suicidal ideation; however, the association was not statistically significant.

Chronic pain is highly prevalent and is associated with increased odds of depression, generalized anxiety, suicidal ideation, and suicidal behavior among working Chilean adults. The findings of this study and those from other populations [54, 55], reinforce the knowledge that chronic pain and associated psychiatric symptoms may not be independent entities but rather comorbid symptoms interacting synergistically [60, 61]. This is consistent with the five most prevalent co-occurring symptom clusters labeled as the “SPADE pentad,” which include chronic pain, anxiety, depression, sleep disturbance, and low energy/fatigue [60]. The co-occurrence of SPADE symptoms negatively affects treatment response and undermines patients’ general health, quality of life, and physical functioning [60]. The recent Institute of Medicine report indicates chronic pain is debilitating and requires immediate, appropriate treatment rather than being sidelined [2]. Given its comorbidity with psychiatric disorders, an interdisciplinary approach that addresses the burdensome co-occurring SPADE symptoms will be most promising for patients with chronic pain and optimize their quality of life and other health outcomes [60].

The results of our study are compatible with the current understanding of the biological mechanism of chronic pain and psychiatric outcomes [50, 62, 63]. Functional imaging studies suggest that this bidirectional relationship is partly attributed to shared neural mechanisms. For instance, participants experiencing lower back pain, fibromyalgia, or other types of chronic pain, show functional imaging alterations in regions responsible for the response to emotional stimuli, like the anterior cingulate cortex and the prefrontal cortex regions [64]. In comparison, participants reporting depressive symptoms showed a shift in the insula region responsible for processing emotional stimuli to a region associated with processing pain in pain-free individuals [64]. Similar evidence has been reported for anxiety and other mood disorders [64].

Some important limitations must be considered when interpreting the results of our study results. First, our study’s cross-sectional data collection design does not allow for determining the temporal relationship between chronic pain and psychiatric outcomes. Second, measures of chronic pain were mainly related to the type of work injury reported by participants at the time of enrollment in the study; thus, we were unable to account for the influence of chronic pain predating the work injury. Furthermore, our use of self-reported data in the exposure, outcome, and other covariates, may have introduced some degree of measurement error. Although we used multivariable logistic regression procedures to adjust for putative confounders, we cannot exclude the possibility of residual confounding. Finally, the participants in this study were injured working adults in Santiago, Chile; thus, the results may not be generalizable to other populations.

## Supplementary Information

**Additional file 1.** Additional information supporting this article can be found in the file: Appendices_SPLENDID_BMC Psychiatry.docx

## Data Availability

Data supporting this study will be provided by the corresponding author on reasonable request.

## References

[1] Goldberg DS, McGee SJ (2011). Pain as a global public health priority. BMC Public Health.

[2] IOM: Institute of Medicine (2011). Relieving pain in America: a blueprint for transforming prevention, care, education, and research.

[3] Patel A, Farquharson R, Carroll D, Moore A, Phillips C, Taylor R, Barden J (2012). The impact and burden of chronic pain in the workplace: a qualitative systematic review. Pain Pract.

[4] Stewart W, Ricci J, Chee E, Morganstein D, Lipton R (2003). Lost productive time and cost due to common pain conditions in the US workforce. Jama.

[5] Means-Christensen AJ, Roy-Byrne PP, Sherbourne CD, Craske MG, Stein MB (2008). Relationships among pain, anxiety, and depression in primary care. Depression and Anxiety.

[6] Chen X, Cheng HG, Huang Y, Liu Z, Luo X (2012). Depression symptoms and chronic pain in the community population in Beijing, China. Psych Res.

[7] Stubbs B, Vancampfort D, Veronese N, Thompson T, Fornaro M, Schofield P, Solmi M, Mugisha J, Carvalho AF, Koyanagi A (2017). Depression and pain: primary data and meta-analysis among 237 952 people across 47 low- and middle-income countries. Psychol Med.

[8] Tsang A, Von Korff M, Lee S, Alonso J, Karam E, Angermeyer MC, Borges GL, Bromet EJ, Demytteneare K, de Girolamo G (2008). Common chronic pain conditions in developed and developing countries: gender and age differences and comorbidity with depression-anxiety disorders. J Pain.

[9] Narasimhan M, Campbell N (2010). A tale of two comorbidities: understanding the neurobiology of depression and pain. Indian J Psychiatry.

[10] Hooley JM, Franklin JC, Nock MK (2014). Chronic pain and suicide: understanding the association. Curr Pain Headache Rep.

[11] Stubbs B (2016). The prevalence and odds of suicidal thoughts, behaviours and deaths among people with painful comorbidities: an updated meta-analysis accounting for publication bias. J Psychiatr Res.

[12] Ilgen MA, Zivin K, McCammon RJ, Valenstein M (2008). Pain and suicidal thoughts, plans and attempts in the United States. Gen Hosp Psychiatry.

[13] Calati R, Courtet P (2016). Is psychotherapy effective for reducing suicide attempt and non-suicidal self-injury rates? Meta-analysis and meta-regression of literature data. J Psychiatr Res.

[14] Roy R, Sommer JL, Bolton JM, El-Gabalawy R (2021). Understanding correlates of suicidality among those with usual pain and discomfort: a Canadian nationally representative study. J Psychosom Res.

[15] Demyttenaere K, Bruffaerts R, Lee S, Posada-Villa J, Kovess V, Angermeyer MC, Levinson D, de Girolamo G, Nakane H, Mneimneh Z (2007). Mental disorders among persons with chronic back or neck pain: results from the world mental health surveys. Pain.

[16] Pereira FG, Franca MH, Paiva MCA, Andrade LH, Viana MC (2017). Prevalence and clinical profile of chronic pain and its association with mental disorders. Rev Saude Publica.

[17] Depintor JD, Bracher ES, Cabral DM, Eluf-Neto J (2016). Prevalence of chronic spinal pain and identification of associated factors in a sample of the population of Sao Paulo, Brazil: cross-sectional study. Sao Paulo Med J.

[18] Fanger P, Azevedo R, Mauro M, Lima D, Gaspar K, Silva V, Nascimento W, Botega N (1992). Depression and suicidal behavior of cancer inpatients: prevalence and associated factors. Rev Assoc Med Bras.

[19] Amaya Arias AC, Bruce A, Herrán D, Martín Arango A, Muñoz K, Abella P (2013). Variables associated with the risk of suicide in patients with chronic pain seen in a hospital outpatient clinic in Bogotá. Colombian Journal of Anesthesiology.

[20] Melzack R (1987). The short-form McGill pain questionnaire. Pain.

[21] Jumbo SU, MacDermid JC, Packham TL, Athwal GS, Faber KJ (2020). Reproducibility: reliability and agreement parameters of the revised short McGill pain questionnaire Version-2 for use in patients with musculoskeletal shoulder pain. Health Qual Life Outcomes.

[22] Nair AS, Diwan S (2020). Pain scores and statistical analysis—the conundrum. Ain-Sham Journ of Anesth.

[23] Kim TK (2017). Practical statistics in pain research. Korean J Pain.

[24] DeCoster J, Gallucci M (2011). Iselin A-MR: best practices for using median splits, artificial categorization, and their continuous alternatives. Journal of Experimental Psychopathology.

[25] Garcia D, MacDonald S, Archer T (2015). Two different approaches to the affective profiles model: median splits (variable-oriented) and cluster analysis (person-oriented). PeerJ.

[26] Iacobucci D, Posavac SS, Kardes FR, Schneider MJ, Popovich DL (2015). Toward a more nuanced understanding of the statistical properties of a median split. J Consum Psychol.

[27] Keilani M, Crevenna R, Dorner TE (2018). Sleep quality in subjects suffering from chronic pain. Wien Klin Wochenschr.

[28] Kitisomprayoonkul W, Klaphajone J, Kovindha A (2006). Thai short-form McGill pain questionnaire. J Med Assoc Thail.

[29] Alharbi HA, Albabtain MA, Alobiad N, Aba Alhasan J, Alruhaimi M, Alnefisah M, Alateeq S, Alghosoon H, Alarfaj SJ, Arafat AA (2020). Pain perception assessment using the short-form McGill pain questionnaire after cardiac surgery. Saudi J Anaesth.

[30] Manea L, Gilbody S, McMillan D (2012). Optimal cut-off score for diagnosing depression with the patient health questionnaire (PHQ-9): a meta-analysis. Cmaj.

[31] Wulsin L, Somoza E, Heck J (2002). The feasibility of using the Spanish PHQ-9 to screen for depression in primary care in Honduras. Primary Care Companion to the Journal of Clinical Psychiatry.

[32] Zhong Q, Gelaye B, Fann JR, Sanchez SE, Williams MA (2014). Cross-cultural validity of the Spanish version of PHQ-9 among pregnant Peruvian women: a Rasch item response theory analysis. J Affect Disord.

[33] Baader M (2012). JL M, Venezian S, Rojas C, Farías R, Fierro-Freixenet C, Backenstrass M, C M: Validación y utilidad de la encuesta PHQ-9 (patient health questionnaire) en el diagnóstico de depresión en pacientes usuarios de atención primaria en Chile. Revista chilena de neuro-psiquiatría.

[34] Spitzer RL, Kroenke K, Williams JBW, Löwe B (2006). A brief measure for assessing generalized anxiety disorder: the GAD-7. Arch Intern Med.

[35] García-Campayo J, Zamorano E, Ruiz M, Pardo A, Pérez-Páramo M, López-Gómez V, Freire O, Rejas J (2010). Cultural adaptation into Spanish of the generalized anxiety disorder-7 (GAD-7) scale as a screening tool. Health Qual Life Outcomes.

[36] Zhong QY, Gelaye B, Zaslavsky AM, Fann JR, Rondon MB, Sanchez SE, Williams MA (2015). Diagnostic validity of the generalized anxiety disorder - 7 (GAD-7) among pregnant women. PLoS One.

[37] Posner K, Brown G, Stanley B, Brent D, Yershova K, Oquendo M, Currier G, Melvin G, Greenhill L, Shen S (2011). The Columbia–Suicide Severity Rating Scale: initial validity and internal consistency findings from three multisite studies with adolescents and adults. Am J Psychiatry.

[38] Conway PM, Erlangsen A, Teasdale TW, Jakobsen IS, Larsen KJ (2017). Predictive validity of the Columbia-suicide severity rating scale for short-term suicidal behavior: a Danish study of adolescents at a high risk of suicide. Arch Suicide Res.

[39] Al-Halabí S, Sáiz P, Burón P, Garrido M, Benabarre A, Jiménez E, Cervilla J, Navarrete M, Díaz-Mesa E, García-Álvarez L (2016). Validation of a Spanish version of the Columbia-suicide severity rating scale (C-SSRS). Rev Psiquiatr Salud Ment.

[40] Serrani Azcurra D (2017). Psychometric validation of the Columbia-suicide severity rating scale in Spanish-speaking adolescents. Colombia médica.

[41] Frank J, Brooker A, DeMaio S, Kerr M, Maetzel A, Shannon H, Sullivan T, Norman R, Wells R (1996). Disability resulting from occupational low back pain. Part II: what do we know about secondary prevention? A review of the scientific evidence on prevention after disability begins. Spine.

[42] Steenstra IA, Munhall C, Irvin E, Oranye N, Passmore S, Van Eerd D, Mahood Q, Hogg-Johnson S (2017). Systematic review of prognostic factors for return to work in workers with sub acute and chronic low Back pain. J Occup Rehabil.

[43] Velez JC, Friedman LE, Barbosa C, Castillo J, Juvinao-Quintero DL, Williams MA, Gelaye B (2022). Evaluating the performance of the pain interference index and the short form McGill pain questionnaire among Chilean injured working adults. PLoS One.

[44] Ratcliffe GE, Enns MW, Belik SL, Sareen J (2008). Chronic pain conditions and suicidal ideation and suicide attempts: an epidemiologic perspective. Clin J Pain.

[45] Milner A, Spittal MJ, Pirkis J, LaMontagne AD (2013). Suicide by occupation: systematic review and meta-analysis. Br J Psychiatry.

[46] Rossom RC, Coleman KJ, Ahmedani BK, Beck A, Johnson E, Oliver M, Simon GE (2017). Suicidal ideation reported on the PHQ9 and risk of suicidal behavior across age groups. J Affect Disord.

[47] Ribeiro JD, Huang X, Fox KR, Franklin JC (2018). Depression and hopelessness as risk factors for suicide ideation, attempts and death: meta-analysis of longitudinal studies. Br J Psychiatry.

[48] Kroenke K, Spitzer R, Williams J, Lowe B (2010). The patient health questionnaire somatic, anxiety, and depressive symptom scales: a systematic review. Gen Hosp Psychiatry.

[49] Kroenke K, Strine T, Spitzer R, Williams J, Berry J, Mokdad A (2009). The PHQ-8 as a measure of current depression in the general population. J Affect Disord.

[50] Mills SEE, Nicolson KP, Smith BH (2019). Chronic pain: a review of its epidemiology and associated factors in population-based studies. Br J Anaesth.

[51] Portenoy RK, Ugarte C, Fuller I, Haas G (2004). Population-based survey of pain in the United States: differences among white, african american, and hispanic subjects. J Pain.

[52] Bilbeny N, Miranda JP, Eberhard ME, Ahumada M, Méndez L, Orellana ME, Cid L, Ritter P, Fernández R (2018). Survey of chronic pain in Chile – prevalence and treatment, impact on mood, daily activities and quality of life. Scand J Pain.

[53] Díaz Cabezas R, Marulanda Mejía F, Sáenz X (2009). Estudio epidemiológico del dolor crónico en Caldas, Colombia (Estudio Dolca). Acta Medica Colombiana.

[54] Sansone RA, Watts DA, Wiederman MW (2014). Pain, pain catastrophizing, and history of intentional overdoses and attempted suicide. Pain Pract.

[55] Braden JB, Sullivan MD (2008). Suicidal thoughts and behavior among adults with self-reported pain conditions in the national comorbidity survey replication. J Pain.

[56] Almeida OP, Draper B, Snowdon J, Lautenschlager NT, Pirkis J, Byrne G, Sim M, Stocks N, Flicker L, Pfaff JJ (2012). Factors associated with suicidal thoughts in a large community study of older adults. Br J Psychiatry.

[57] Juurlink D, Herrmann N, Szalai J, Kopp A, Redelmeier D (2004). Medical illness and the risk of suicide in the elderly. Arch Intern Med.

[58] Kikuchi N, Ohmori-Matsuda K, Shimazu T, Sone T, Kakizaki M, Nakaya N, Kuriyama S, Tsuji I (2009). Pain and risk of completed suicide in Japanese men: a population-based cohort study in Japan (Ohsaki cohort study). J Pain Symptom Manag.

[59] Srivastava MK, Sahoo RN, Ghotekar LH (2004). SrihariDutta, Danabalan M, Dutta TK, das AK: risk factors associated with attempted suicide: a case control study. Indian J Psychiatry.

[60] Davis LL, Kroenke K, Monahan P, Kean J, Stump TE (2016). The SPADE symptom cluster in primary care patients with chronic pain. Clin J Pain.

[61] Aktas A (2013). Cancer symptom clusters: current concepts and controversies. Curr Opin Support Palliat Care.

[62] van der Windt D, Croft P, Penninx B (2002). Neck and upper limb pain: more pain is associated with psychological distress and consultation rate in primary care. J Rheumatol.

[63] Breivik H, Collett B, Ventafridda V, Cohen R, Gallacher D (2006). Survey of chronic pain in Europe: prevalence, impact on daily life, and treatment. Eur J Pain.

[64] Hooten WM (2016). Chronic pain and mental health disorders: shared neural mechanisms, epidemiology, and treatment. Mayo Clin Proc.

