# Supplementary material for: Pain, mood, and suicidal behavior among injured working adults in Chile
Source: BMC Psychiatry. 2022 Dec 6;22:766. doi: 10.1186/s12888-022-04391-3 (PMC9724445; doi:10.1186/s12888-022-04391-3)
Supplement: Supplementary file 1 — Additional file 1. Additional information supporting this article can be found in the file: Appendices_SPLENDID_BMC Psychiatry.docx [file 12888_2022_4391_MOESM1_ESM.docx]

# Appendices

# Supplementary Table 1. Association of pain with depression, anxiety, and suicidal behavior: estimated regression coefficients (OR) and 95% CI.

|  | **Unadjusted model**  **beta (95% CI)** | **Adjusted^a^**  **beta (95% CI)** | **Adjusted^b^**  **beta (95% CI)** | **Adjusted^c^**  **beta (95% CI)** |
| --- | --- | --- | --- | --- |
|  |  |  |  |  |
| Depression or anxiety |  |  |  |  |
| No | Reference | Reference | Reference | - |
| Yes | 1.10(1.09-1.12) | 1.09(1.08-1.11) | 1.09(1.08,1.11) | - |
| Depression and anxiety |  |  |  |  |
| No | Reference | Reference | Reference | - |
| Depression only (PHQ-9) | 1.07(1.05-1.09) | 1.07(1.05-1.08) | 1.07(1.05,1.08) | - |
| Anxiety only (GAD-7) | 1.08(1.05-1.11) | 1.08(1.05-1.11) | 1.08(1.04-1.11) | - |
| Both depression and anxiety | 1.14(1.12-1.16) | 1.13(1.12-1.15) | 1.13(1.12-1.15) | - |
| ***Suicidal Behaviors*** |  |  |  |  |
| Suicidal ideation |  |  |  |  |
| No suicidal ideation | Reference | Reference | Reference | Reference |
| Suicidal ideation | 1.10(1.09-1.12) | 1.09(1.08-1.12) | 1.09(1.08-1.11) | 1.07(1.06-1.09) |
| Suicidal behavior |  |  |  |  |
| No suicidal behavior | Reference | Reference | Reference | Reference |
| Suicidal behavior | 1.08(1.05-1.10) | 1.07(1.05-1.10) | 1.06(1.03-1.09) | 1.03(1-1.06) |

Depression and generalized anxiety symptoms were assessed based on thresholds of the PHQ-9 and the GAD-7 scores, respectively. Suicidal behaviors were assessed using the C-SSRS scale. Effect estimates are interpreted as the odds of depression, anxiety or suicidal behavior, per unit increase in the continuous scale of the SF-MPG score.

^a^ Adjusted for age (continuous), sex (male/female), marital status (married/single/previously married)

^b^ Further adjusted for type of workplace (categories), type of injury (categories), and time since accident (categories)

^c^ Further adjusted for type of workplace (categories), type of injury (categories), and time since accident (categories), and depression (PHQ-8)

Supplementary Table 2. Association of sensory pain subscale with depression, anxiety, and suicidal behavior among a population of injured working adults in Santiago, Chile (N=1,946).

| **Depression, anxiety and suicidal ideation** | **Low sensory pain**  **(N = 1,062)** | |  | | **High sensory pain**  **(N = 884)** | | | | | |
| --- | --- | --- | --- | --- | --- | --- | --- | --- | --- | --- |
|  | **n** | **%** | |  | **n** | **%** | **Unadjusted OR**  **(95% CI)** | **Adjusted OR**  **(95% CI) ^a^** | **Adjusted OR**  **(95% CI) ^b^** | **Adjusted OR**  **(95% CI) ^c^** |
|  |  |  | |  |  |  |  |  |  |  |
| Depression ***^b^*** or anxiety |  |  | |  |  |  |  |  |  |  |
| No | 775 | 73.0 | |  | 356 | 40.3 | Reference | Reference | Reference | - |
| Yes | 287 | 27.0 | |  | 528 | 59.7 | 1.13(1.11-1.15) | 1.13(1.11-1.15) | 1.13(1.11-1.15) | - |
| Depression or anxiety |  |  | |  |  |  |  |  |  |  |
| No | 775 | 73.0 | |  | 356 | 40.3 | Reference | Reference | Reference | - |
| Depression only (PHQ-9) | 153 | 14.4 | |  | 199 | 22.5 | 1.09(1.06-1.11) | 1.08(1.06-1.11) | 1.08(1.06-1.11) | - |
| Anxiety only (GAD-7) | 36 | 3.4 | |  | 35 | 4.0 | 1.09(1.06-1.14) | 1.09(1.05-1.14) | 1.09(1.05-1.14) | - |
| Both depression and anxiety | 98 | 9.2 | |  | 294 | 33.3 | 1.18(1.16-1.20) | 1.17(1.15-1.20) | 1.17(1.15-1.20) | - |
| ***Suicidal Ideation*** |  |  | |  |  |  |  |  |  |  |
| Suicidal ideation |  |  | |  |  |  |  |  |  |  |
| No suicidal ideation | 983 | 92.6 | |  | 702 | 79.4 | Reference | Reference | Reference | Reference |
| Suicidal ideation | 79 | 7.4 | |  | 182 | 20.6 | 1.13(1.11-1.15) | 1.13(1.10-1.15) | 1.11(1.09-1.14) | 1.09(1.07-1.11) |
| Suicidal behavior |  |  | |  |  |  |  |  |  |  |
| No suicidal behavior | 1050 | 98.9 | |  | 849 | 96.0 | Reference | Reference | Reference | Reference |
| Suicidal behavior | 12 | 1.1 | |  | 35 | 4.0 | 1.09(1.05-1.13) | 1.09(1.05-1.13) | 1.06(1.02-1.10) | 1.03(0.99-1.07) |

^a^ Adjusted for age (continuous), sex (male, female), marital status (married/living with a partner, single, previously married).

^b^ Further adjusted for type of workplace (categories), type of injury (categories), and time since the accident (categories).

^c^ Further adjusted for type of workplace (categories), type of injury (categories), and time since the accident (categories), and depression (PHQ-8)

**Supplementary Table 3.** Association of affective pain subscale with depression, anxiety, and suicidal behavior among a population of injured working adults in Santiago, Chile (N=1,946).

| **Mood disorders and suicidal behaviors** | **Low affective pain**  **(N = 1,184)** | |  | | **High affective pain**  **(N = 762)** | | | | | |
| --- | --- | --- | --- | --- | --- | --- | --- | --- | --- | --- |
|  | **n** | **%** | |  | **n** | **%** | **Unadjusted OR**  **(95% CI)** | **Adjusted OR**  **(95% CI) ^a^** | **Adjusted OR**  **(95% CI) ^b^** | **Adjusted OR**  **(95% CI) ^c^** |
|  |  |  | |  |  |  |  |  |  |  |
| Depression ***^b^*** or anxiety |  |  | |  |  |  |  |  |  |  |
| No | 842 | 71.1 | |  | 289 | 37.9 | Reference | Reference | Reference | - |
| Yes | 342 | 28.9 | |  | 473 | 62.1 | 1.25(1.26-1.36) | 1.29(1.24-1.34) | 1.29(1.24-1.34) | - |
| Depression or anxiety |  |  | |  |  |  |  |  |  |  |
| No | 842 | 71.1 | |  | 289 | 37.9 | Reference | Reference | Reference | - |
| Depression only (PHQ-9) | 189 | 16.0 | |  | 163 | 21.4 | 1.19(1.13-1.24) | 1.17(1.12-1.23) | 1.17(1.12-1.23) | - |
| Anxiety only (GAD-7) | 34 | 2.9 | |  | 37 | 4.9 | 1.22(1.13-1.33) | 1.21(1.11-1.32) | 1.21(1.11-1.32) | - |
| Both depression and anxiety | 119 | 10.1 | |  | 273 | 35.8 | 1.43(1.36-1.49) | 1.40(1.34-1.47) | 1.41(1.34-1.47) | - |
| ***Suicidal Ideation*** |  |  | |  |  |  |  |  |  |  |
| Suicidal ideation |  |  | |  |  |  |  |  |  |  |
| No suicidal ideation | 1107 | 93.5 | |  | 578 | 75.9 | Reference | Reference | Reference | Reference |
| Suicidal ideation | 77 | 6.5 | |  | 184 | 24.1 | 1.35(1.29-1.40) | 1.33(1.28-1.39) | 1.30(1.25-1.36) | 1.24(1.18-1.29) |
| Suicidal behavior |  |  | |  |  |  |  |  |  |  |
| No suicidal behavior | 1173 | 99.1 | |  | 726 | 95.3 | Reference | Reference | Reference | Reference |
| Suicidal behavior | 11 | 0.9 | |  | 36 | 4.7 | 1.29(1.2-1.39) | 1.29(1.19-1.39) | 1.23(1.14-1.33) | 1.16(1.06-1.26) |

^a^ Adjusted for age (continuous), sex (male, female), marital status (married/living with a partner, single, previously married)

^b^ Further adjusted for type of workplace (categories), type of injury (categories), and time since the accident (categories)

^c^ Further adjusted for type of workplace (categories), type of injury (categories), and time since the accident (categories), and depression (PHQ-8)
